# High-Dose Epidermal Radionuclide Therapy with ^188^ Re (Rhenium) Resin in a Patient with Multiple Actinic Keratoses

**DOI:** 10.1055/s-0044-1788075

**Published:** 2024-07-04

**Authors:** Siroos Mirzaei, Rainer Kunstfeld

**Affiliations:** 1 Department of Nuclear Medicine with PET-Centre, Clinic Ottakring, Vienna, Austria; 2 University Department for Dermatology, Medical University Vienna, Vienna, Austria

**Keywords:** actinic keratosis, epidermal therapy, radionuclide therapy, Rhenium-188, skin cancer

## Abstract

**Aim** High-dose epidermal radionuclide therapy using a nonsealed ^188^ Re (Rhenium) resin is an alternative treatment option for nonmelanoma skin cancer. In this case study, we present the possible use of this therapy in a patient with multiple actinic keratosis (AK), which is a precancer of the skin.

**Methods** A 55-year-old male was presented in our department with multiple AK, located on the cheek, temporal, and frontal area, with 1, 1, 2.1, and 2.5 cm ^2^ surface.

Applied activity was 80, 80, 167, and 168 MBq ^188^ Re with a target absorbed dose for each lesion 35 Gy at 1 mm. The treatment was well tolerated.

**Results** At 17 months’ follow-up, all treated area showed complete remission. There were no side effects, except mild focal hypopigmentation.

**Conclusion** This case demonstrates the high potential of epidermal radionuclide therapy with a nonsealed ^188^ Re as a noninvasive, effective, and well-tolerated therapy option for patients with multiple AK, when surgery is difficult to perform or not recommended of other reasons.

## Introduction

Actinic keratoses, also known as senile or solar keratoses, are benign intraepithelial neoplasms. Irregular, red, scaly papules, or plaques may appear on sun-exposed areas of the body in people with actinic keratosis. As actinic keratosis can potentially progress to invasive squamous cell carcinoma, early recognition and implementation of a treatment plan is crucial. 1

Actinic keratoses are primarily caused by the cumulative effect of ultraviolet radiation on the skin. This occurs over a lifetime of sun exposure. 2

Treatment options for actinic keratosis can be categorized into lesional and field-directed therapies. The treatment mantra often associated with actinic keratosis is “no pain, no gain,” implying that effective treatment may involve some discomfort or side effects. 1

Lesion-directed therapies focus on treating individual actinic keratoses. The standard options include cryotherapy, curettage, or surgical excision of the lesion. These therapies are effective for targeting specific visible lesions.

On the other hand, field-directed therapies have the advantage of being able to treat multiple, widespread, and subclinical actinic keratoses within an area of chronic sun damage. The aim of these therapies is the treatment of the entire affected area of skin rather than the treatment of individual lesions. Field-directed therapies may include topical medications (chemotherapeutic creams or immunomodulators), light-based therapies such as photodynamic therapy, or laser resurfacing. It is important to recognize that no treatment for actinic keratosis is completely risk free. Some of the most common potential adverse effects are pain, inflammation, problems with healing, changes in pigmentation, and scarring. 1

Recurrence of actinic keratosis and the need for multiple treatments are common occurrences. The healing process may range from days to weeks, depending on the location and number of lesions treated.

We present the case of a patient with multiple actinic keratoses on the face. The patient underwent epidermal therapy with ^188^ Rhenium ( ^188^ Re).

## Patient and Methods

**Fig. 1:**
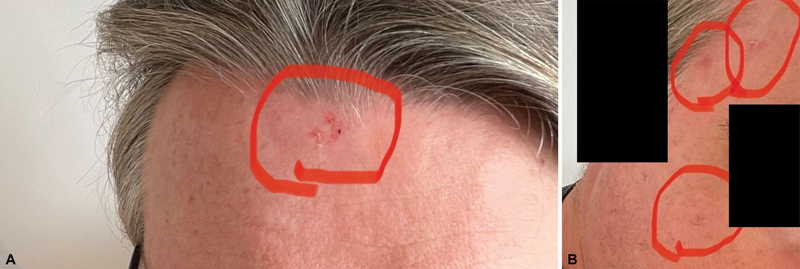
Actinic keratoses on the face prior to treatment in the frontal region ( **A** ) and in the cheek and temporal region ( **B** ).

A 55-year-old man was presented to our department with multiple actinic keratoses located on the frontal areas ( Fig. 1A ), cheek, and temporal areas ( Fig. 1B ) with a surface area of 1, 1, 2.1, and 2.5 cm ^2^ .

A written informed consent for the treatment was obtained from the patient. We applied the activity of 80, 80, 167, and 168 MBq ^188^ Re (Rhenium-SCT, Oncobeta GmbH, Munich, Germany) with a target absorbed dose of 35 Gy at 1 mm for each lesion. During the treatment and the following weeks, the treatment was well tolerated.

## Results

**Fig. 2:**
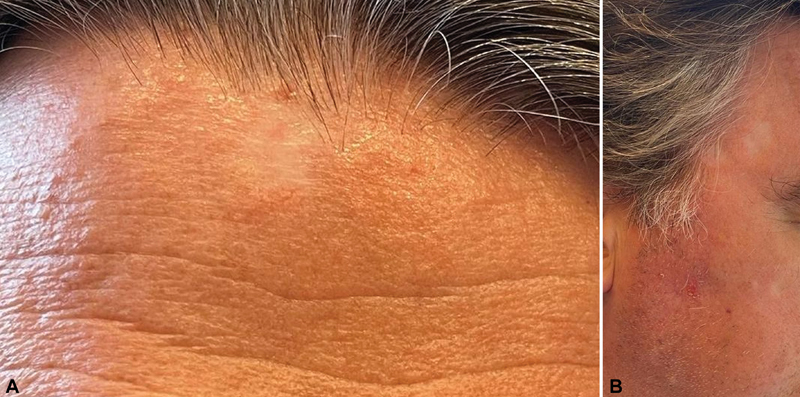
Remission 17 months after receiving treatment with ^188^ Rhenium ( **A** , **B** ).

At 18 months' follow-up, all treated areas showed complete remission ( Fig. 2A, B ). No side effects, except mild focal hypopigmentation, were observed. There was no need for further medication after treatment.

## Discussion

Few papers have been published on the use of epidermal radionuclide therapy for nonmelanoma skin cancer using ^188^ Re. 3 4 5

High-dose epidermal radionuclide therapy using an unsealed ^188^ Re resin is a new treatment option that allows radioactivity to be delivered as close as possible to the surface of skin lesions. This technique is based on the property of ^188^ Re to release a high-energy, emitting 85% β radiation (beta 2.2 MeV). It is known that ^188^ Re releases 92% of its energy within 2-mm depth in the skin. 6

Treatment with ^188^ Re is a painless and fast technique that can be tailored to the patient in a single session and is likely to provide a better aesthetic result than surgery. The results of this study are very promising and the treatment is well tolerated with only minor side effects, such as hypopigmentation of the treated area in this case ( Fig. 2A, B ). The technique can therefore be proposed as an alternative therapeutic choice for actinic keratoses at sites unsuitable for surgical resection.

## Conclusion

This case demonstrates the high potential of epidermal radionuclide therapy with ^188^ Re as a noninvasive, effective, and well-tolerated treatment option for patients with multiple actinic keratoses in whom surgery is difficult to perform or is not recommended for other reasons.
